# Vocal Complexity Constrains the Dear Enemy Effect: A Comparative Study of Coal Tits and Green‐Backed Tits

**DOI:** 10.1002/ece3.72918

**Published:** 2026-01-09

**Authors:** Lin Zhao, Fangfang Zhang, Jianping Liu, Wei Liang

**Affiliations:** ^1^ Ministry of Education Key Laboratory for Ecology of Tropical Islands, Key Laboratory of Tropical Animal and Plant Ecology of Hainan Province, College of Life Sciences Hainan Normal University Haikou China; ^2^ College of Biological Sciences and Engineering North Minzu University Yinchuan China

**Keywords:** bioacoustics, dear enemy effect, individual recognition, neighbor–stranger discrimination, song repertoire complexity, territorial aggression

## Abstract

The “dear enemy effect,” wherein territorial animals exhibit reduced aggression toward familiar neighbors compared to strangers, is a widespread strategy to minimize energy expenditure on territory defense. However, whether and how this behavioral capacity varies across with differing vocal complexity remains poorly unclear. We investigated neighbor–stranger discrimination (NSD) in two sympatric tit species that exhibit a stark contrast in song repertoire complexity: coal tits ( *Periparus ater* ) and green‐backed tits ( *Parus monticolus* ). Acoustic analysis revealed that coal tits possessed a large population‐level song‐type diversity (19 distinct song types) and, crucially, a significantly larger individual syllable repertoire size compared to green‐backed tits (5 song types). Playback experiments showed that coal tits exhibited a robust “dear enemy” effect, responding to strangers with significantly closer approach distance and higher flight frequencies near the nest. In contrast, green‐backed tits showed uniformly low and undifferentiated responses toward both playbacks of familiar neighbors and strangers, indicating a lack of discrimination. This interspecific divergence was underpinned differences in individual repertoire size and population‐level acoustic diversity, with green‐backed tits exhibiting higher vocal similarity among individuals. These results demonstrate that the capacity for fine‐scale NSD is not universal and suggest that constrained vocal systems—characterized by minimal individual repertoires and high acoustic similarity among individuals—may limit the potential for vocal individual recognition, thereby favoring alternative territorial strategies.

## Graphical abstract

This study investigated neighbor–stranger discrimination in two sympatric tit species exhibiting contrasts in song repertoire complexity: coal tits ( *Periparus ater* ) and green‐backed tits ( *Parus monticolus* ). Coal tits exhibited robust discrimination, responding with significantly greater aggression to strangers. In contrast, green‐backed tits showed a lack of discrimination. The dear enemy effect is not universal and that vocal repertoire complexity plays a critical role in shaping individual recognition abilities.

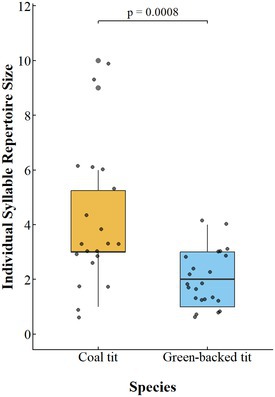

## 1 Introduction

In territorial animals, neighbor–stranger discrimination (NSD) is a crucial behavioral strategy that minimizes energy and time expenditure on territory defense, thereby optimizing resource allocation for survival and reproduction (Temeles 1994; Carlson, Healy, and Templeton 2020; Carlson, Kelly, and Couzin 2020). The classic “dear enemy” effect describes a scenario wherein territory owners exhibit less aggression toward familiar neighbors than toward unfamiliar strangers, representing a tradeoff between threat assessment and energy conservation (Fisher 1954). Conversely, the “nasty neighbor” effect refers to heightened aggression toward neighbors, potentially arising from intense local resource competition or individual conflicts (Temeles 1994).

Two primary hypotheses explain these behavioral patterns: The threat hypothesis posits that behavioral responses are calibrated to the relative threat level posed by intruders, leading to stronger aggression toward unpredictable strangers (Temeles 1994), while the familiarity hypothesis emphasizes that repeated nonaggressive interactions with familiar neighbors reduce information acquisition costs, allowing resources reallocation to other vital activities (Temeles 1994; Briefer, Rybak, and Aubin 2008). Empirical evidence predominantly supports the threat hypothesis, indicating that stranger‐associated uncertainty elicits stronger defensive responses across diverse taxa (Carlson, Healy, and Templeton 2020; Carlson, Kelly, and Couzin 2020), including invertebrates (Fogo et al. 2019), amphibians (Bee and Gerhardt 2001), fish (Leiser 2003), reptiles (Ventura et al. 2023), birds (Radford 2005; Moskát et al. 2017; Jin et al. 2021; Niśkiewicz et al. 2024), and mammals (Holzmann and Córdoba 2024). The adaptive significance of this mechanism lies in its efficiency: by minimizing unnecessary energy expenditure on assessing familiar neighbors, animals can optimize their investment in territory defense while maximizing energy available for critical survival and reproductive tasks (Temeles 1994; Briefer, Rybak, and Aubin 2008).

NSD has been extensively studied in avian species, encompassing both oscine and nonoscine passerines. Accumulating evidence underscores that its expression shows remarkable plasticity and context dependency, modulated by key ecological and social variables such as territory stability, breeding phase and mate availability (Briefer, Rybak, and Aubin 2008; Budka and Osiejuk 2013; Moser‐Purdy et al. 2017; Amorim et al. 2022; Jin et al. 2021). For instance, great tits ( *Parus major* ) and Eurasian skylark ( *Alauda arvensis* ) exhibit robust dear enemy effect during stable breeding periods but not during territorial establishment phases (Briefer, Rybak, and Aubin 2008; Jin et al. 2021), while male song sparrows ( *Melospiza melodia* ) intensify aggression toward strangers during mate fertility periods (Moser‐Purdy et al. 2017). These findings, suggesting a dynamic adjustment of territorial aggression in response to reproductive context, collectively indicate that NSD is not a stereotypic behavior but a facultative strategy shaped by immediate socioecological demands.

Bird vocalizations serve as primary acoustic signals for individual recognition exhibiting remarkable interspecific diversity. Traditional hypotheses suggested large song repertoires might impede individual discrimination due to reduced shared acoustic elements and increased cognitive load (Falls and d'Agincourt 1981; McGregor and Avery 1986). However, emerging evidence indicates that species with complex repertoires like Eurasian skylark and red‐eyed vireos ( *Vireo olivaceus* ), demonstrate enhanced discrimination capabilities through increased individualization in song encoding (Briefer, Rybak, and Aubin 2008; Moser‐Purdy and Mennill 2016). Notably, empirical investigations of NSD in species with simplified vocal repertoires remain notably scarce (Stoddard 1996; Leclerc et al. 2025), leaving unclear whether vocal simplicity constrains individual recognition.

The relationship between avian song complexity and individual recognition capacity remains a subject of theoretical debate. Early hypotheses posited that large, complex repertoires might impede individual recognition by reducing shared, predictable song elements, thereby increasing the cognitive load for receivers (Falls and d'Agincourt 1981; McGregor and Avery 1986). Contrary to this view, accumulating evidence indicates that species with complex songs, such as the Eurasian skylark ( *Alauda arvensis* ) and the red‐eyed vireo ( *Vireo olivaceus* ), can achieve sophisticated discrimination, likely by encoding individual signatures across diverse song types or through combinatorial syntax (Briefer, Aubin, et al. 2008; Moser‐Purdy and Mennill 2016). Recent work on the pied flycatcher ( *Ficedula hypoleuca* ) further confirms that high song complexity does not preclude fine‐scale acoustic discrimination, even at the population level (Gallego‐Abenza et al. 2025).

Conversely, effective neighbor–stranger discrimination (NSD) is also well‐documented in species with small, stereotyped repertoires (e.g., Ręk and Osiejuk 2011; Osiejuk 2014; Niśkiewicz et al. 2024). These species appear to rely on subtle within‐song‐type variation in spectral or temporal features for individual recognition. Notably, this capacity extends beyond oscine passerines, as evidenced by the rufous hornero ( *Furnarius rufus* , a suboscine), which exhibits a robust “dear enemy” effect that is stable across different territory sizes and levels of human disturbance (Amorim et al. 2023). Collectively, these studies demonstrate that repertoire size is merely one dimension of acoustic complexity, and its role in facilitating or constraining NSD is not straightforward. Therefore, to disentangle the specific effects of vocal complexity from confounding ecological and phylogenetic factors, direct comparisons between sympatric, ecologically similar, and closely related species that exhibit divergent repertoire architectures are crucial—yet such studies remain strikingly rare. Tit species (family Paridae) are able to recognize and respond to each other's songs (Berlusconi, Castiglione, Wauters, et al. 2025). While most tit species behaved more aggressively against a conspecific intruder playback stimulus, a recent study highlighted extremely coherent patterns in the response to heterospecific stimuli within each group of tit species, with “broadleaf tits” responding more strongly toward territorial songs compared to those from different habitats, while this was not the case for “conifer tits” (Berlusconi, Castiglione, Clerici, et al. 2025).

The coal tit ( *Periparus ater* ) and the green‐backed tit ( *Parus monticolus* ) constitute an ideal comparative model for investigating this question. These sympatric, cavity‐nesting passerines share phylogenetic relationships and ecological niche (Johansson et al. 2013) yet exhibit striking vocal repertoire differences. At the population level, coal tits possess a diverse range of song types (Tietze et al. 2011), whereas green‐backed tits exhibit a very limited set. More importantly, at the individual level, coal tits sing with complex, multielement combinatorial patterns, potentially underpinning their role as “community informant” in mixed‐species flocks (Carlson, Healy, and Templeton 2020; Carlson, Kelly, and Couzin 2020). Conversely, green‐backed tits produce simple, highly stereotyped songs, typically formed by repeating single elements (Liao et al. 2007). This contrast provides a controlled context to examine the link between vocal signal complexity and social discrimination abilities, while minimizing confounding ecological and phylogenetic factors.

This study employs playback experiments to compare the two species' behavioral responses to conspecific territorial songs from familiar neighbors versus strangers. It aims to: (1) test whether both species exhibit differential aggression consistent with NSD; (2) determine if response patterns align with the dear enemy or nasty neighbor effect; and (3) explore whether interspecific differences in NSD correlate with quantified differences in song repertoire complexity at both individual and population levels. We predict coal tits will exhibit robust discrimination, while green‐backed tits may show undifferentiated responses, potentially reflecting a constraint imposed by their simpler vocal system.

## 2 Materials and Methods

### 2.1 Study Area

This study was conducted in the Liupanshan National Nature Reserve (35°15′‐35°41′ N, 106°09′‐106°30′ E), Ningxia Hui Autonomous Region, northwestern China. The reserve covers approximately 67,800 ha and features an elevation range of 1935 to 2371 m above sea level with forest coverage exceeding 70%. The climate is mid‐temperate, transitioning semi‐humid to semi‐arid, characterized by a mean annual temperature of 5.8°C and average annual precipitation ranging from 600–800 mm. Vegetation is diverse, primarily consisting of secondary coniferous forest, broadleaf mixed forests, and meadow grasslands. Key tree species include the Chinese red pine ( *Pinus tabuliformis* ) and Asian white birch ( *Betula platyphylla* var. *japonica* ) (Shi et al. 2009).

### 2.2 Vocal Recording and Analysis

#### 2.2.1 Recording Protocol

A total of 374 artificial nest boxes were installed at the study site beginning in the 2021 breeding season, with coal tits and green‐backed tits being the two most common bird species breeding in these nest boxes (Liu et al. 2024).

Vocal recordings were conducted during the 2024 breeding season (April—July). Territoiry songs of conspecific males were recorded 1–3 days before the corresponding playback trials using a Sony PCM‐A10 digital recorder (sampling rate: 48 kHz; resolution:24‐bit; frequency response: 40 Hz‐21 kHz ± 1 dB; max input SPL: 123 dB; dynamic range: 120 dB A‐weighted; Puswal and Liang 2024).

All recordings were obtained from males that occupied active nests with stable breeding status. Recordings were made between 06:00 and 14:00 (Beijing Time, UCT + 8) during the incubation and nestling stages (5–8 days post‐hatching). Each recording session lasted 10–60 min, depending on the focal male's vocal activity. The recorder was positioned on top of the nest box, 0.5–3 m from the subject male.

#### 2.2.2 Syllable and Song Type Identification

Song types were classified acoustically based on note composition and syntactic structure (following Tietze et al. 2011), using Praat version 6.4.10. A syllable was defined as the smallest discrete acoustic unit bounded by silent intervals > 50 ms (for coal tits) and > 100 ms (for green‐backed tits). Consistent syllable composition and temporal patterning were then used to categorize distinct song types.

**FIGURE 1:**
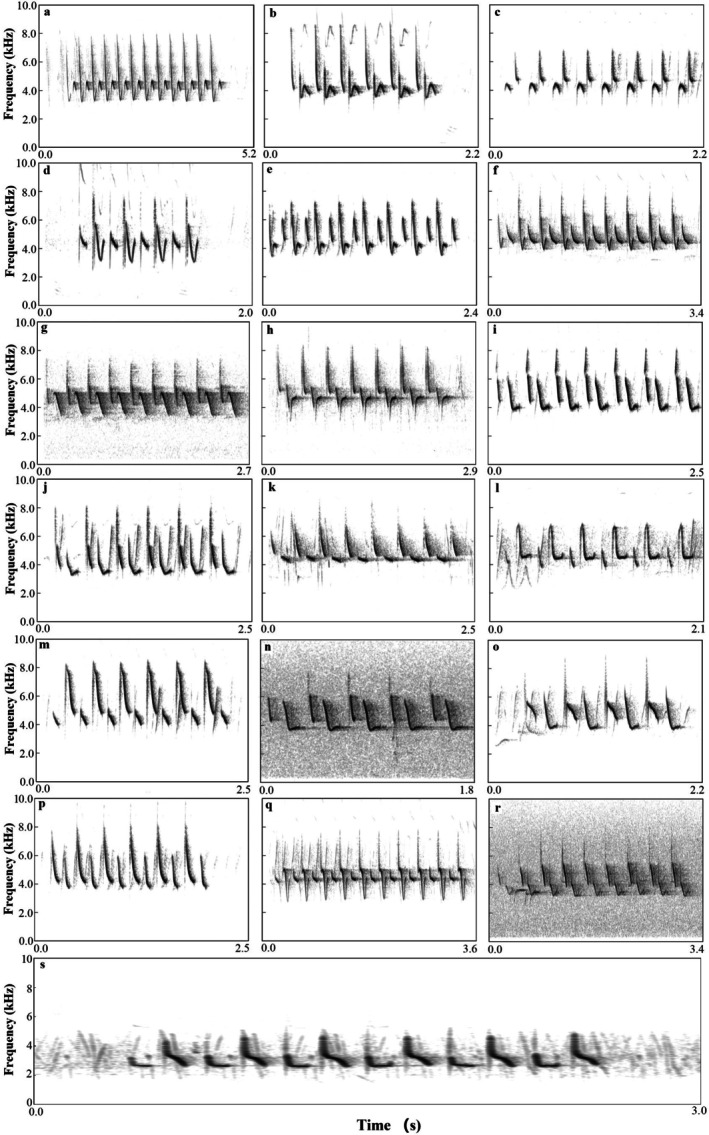
Representative song types in the coal tit repertoire. Spectrogram illustrate the 19 distinct song types (labeled a–s) identified at the population level. Each song type is characterized by unique syllable, and every syllable is composed of two frequency‐modulated notes. This figure provides the acoustic basis for classification criteria used in this study.

**FIGURE 2:**
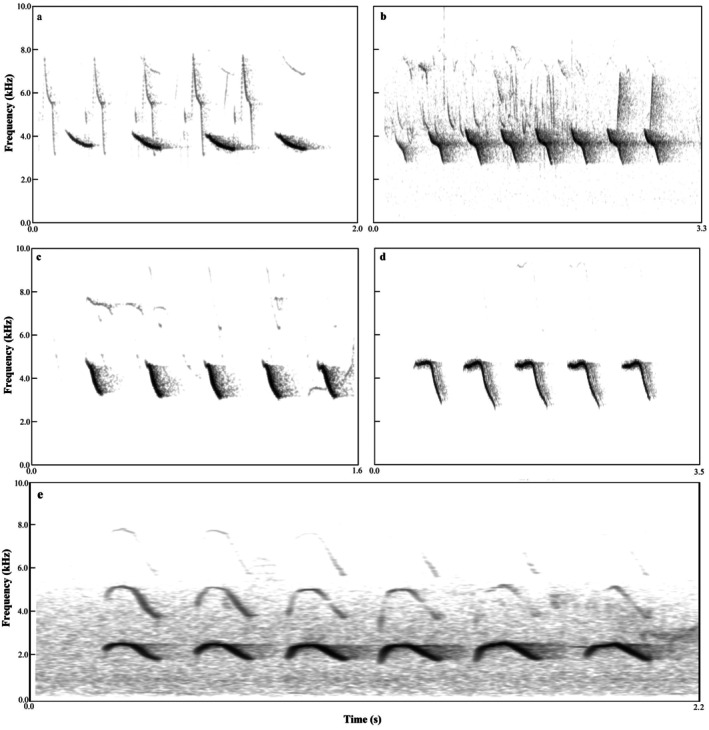
Representative song types in the green‐backed tit repertoire. Spectrogram show the five distinct song types (labeled a–e) identified at the population level. Each song type corresponds to a single, stereotyped syllable (functionally equivalent to a single note in this species), and complete vocalizations are formed by repeating this syllable.

For coal tits, we identified 19 distinct song types. Each type was characterized by a unique syllable, and every syllable consisted of two notes, yielding 19 syllable types in total (labeled a–s, Figure 1). In contrast, green‐backed tits produced only 5 distinct song types. Each song type corresponded to s single syllable, and each syllable (functionally equivalent in this species), resulting in 5 syllable types (labeled a–e, Figure 2).

#### 2.2.3 Individual Repertoire Size Assessment and Population Level Diversity

For each individual male, we calculated the syllable repertoire size as the total number of distinct syllable types produced across all recording sessions. To exclude rare or aberrant vocalizations, only syllables that appeared in at least two separate song phrases were included. Repertoire sizes were compared between species using a nonparametric Wilcoxon rank‐sum test, following confirmation of non‐normal data distribution via the Shapiro–Wilk test.

To quantify acoustic diversity at the population level, Simpson's diversity index (1 − λ) was computed for each species based on syllable type usage across individuals (Jenkins 1978; Podos and Nowicki 2004). To assess individual distinctiveness, Jaccard similarity coefficients were calculated for all pairwise combinations within each species, comparing the sets of syllable types used by each individual (Real and Vargas 1996; Chao et al. 2005).

#### 2.2.4 Playback Stimuli Preparation

All recordings were processed and assembled into playback stimuli using Audacity 3.5.1 (Audacity Team, USA, 2024; https://www.audacityteam.org). Audio files were standardized to a 48 kHz sampling rate and 24‐bit depth, and peak amplitude was normalized to −6 dB to ensure consistent playback levels across all trials.

#### 2.2.5 Playback Stimulus Construction

For coal tits, each 2‐min playback stimulus was created by selecting a single, continuous 2‐min segment from the natural song recordings of a stimulus male. This ensured the stimulus preserved an uninterrupted, natural sequence of song types as produced in a real song bout.

For green‐backed tits, due to species' shorter singing bouts, a 2‐min stimulus was compiled by seamlessly concatenating multiple natural song segments (3–5 segments) from the same male. Care was taken during editing to maintain natural intervals between phrases and to preserve the original sequential order of song types within and across segments. This method aimed to present a representative and ecologically valid sample of the individual's vocal repertoire.

For both species, this approach of using unedited or minimally edited natural recordings as stimuli was chosen to maintain the ecological validity of song sequences, including natural variations in song‐type order, rhythm, and phrase timing. All final stimuli were calibrated to maintain species‐specific natural song rates (coal tits: ~15.7 phrases/min; green‐backed tits: ~11.6 phrases/min) and standardized to −6 dB peak amplitude.

For each experimental trial, one stimulus was randomly selected from the pool of prepared tracks corresponding to the treatment (familiar neighbor or stranger).

#### 2.2.6 Assessment of Potential Sequence Confounds

We acknowledge that natural song sequences vary in length, syllable repertoire, and sequential organization. To ensure that such variation did not systematically differ between our experimental treatments (Familiar neighbor vs. Stranger) and thus confound behavioral responses, we quantified two key structural features for each playback stimulus: the sequence length (number of songs) and the number of distinct syllable types (repertoire units) used. For both coal tits and green‐backed tits, paired *t*‐tests confirmed no significant differences between familiar neighbor and stranger stimuli in either sequence length (coal tit: *t* = 0.571, *p* = 0.575; green‐backed tit: *t* = −0.096, *p* = 0.924) or the number of syllable types used (coal tit: *t* = 0, *p* = 1; green‐backed tit: *t* = −1.438, *p* = 0.165). This confirms that differences in song sequence structure were not a confounding variable in our experiment.

#### 2.2.7 Playback Experimental Design

Playback trials commenced after confirming that a nest was active and occupied by a focal male. We simulated territorial intruders by broadcasting conspecific songs from familiar neighbors or a stranger, following a randomized and counterbalanced design.

Acoustic stimuli were broadcast using a Bogasing‐M10 Bluetooth speaker (frequency response: 80–16 kHz) connected to vivo X100 smartphone (Vivo Communication Technology Co. Ltd., Dongguan, China). The Vivo X100 features a high‐resolution audio playback system that supports 48 kHz sampling rate and 24‐bit depth. Its built‐in Hi‐Fi audio chip (Cirrus Logic CS47L15) ensures low‐distortion digital‐to‐analog conversion, providing high‐fidelity audio output suitable for field playback experiments. Playback amplitude was calibrated to 75–80 dB SPL at a distance of one meter using a Class 2 sound level meter (Delixi electric DSM‐D1) with A weighting. This sound pressure level falls within the natural amplitude range of territorial songs for these species.

The speaker in which the two stimulus types (familiar neighbor vs. stranger) were presented was fully randomized for each subject. Each trial consisted of continuous 2‐min playback, immediately followed by a 2‐min silent observation period. Consecutive trials for the same individual were separated by a minimum interval of 20 min to minimize carry‐over effects and habituation (York and Davies 2017; Yu et al. 2019).

Familiar neighbor stimuli were derived from recordings of males whose nest boxes were located 20–100 m from the subject's nest. To ensure acoustic unfamiliarity, stranger stimuli were derived from recordings of males in a separate study area located more than 5 km from all subject territories.

#### 2.2.8 Experimental Subject Criteria and Trial Initiation

All focal males were paired. Trials were conducted during the incubation or nestling stage (5–8 days posthatching). An “active nest” was defined as one where either: (1) both adults delivered food to nestlings on ≥ 2 consecutive days prior to trial (nestling stage), or (2) the female was observed incubating for > 30 min and eggs were confirmed warm within 1–2 h before trial (incubation stage). Playback began only when the focal male was absent from the observer's view around the nest, ensuring it was neither in close‐range defense nor vocalizing. Trials were conducted on clear or overcast days, without rain or strong wind.

#### 2.2.9 Behavioral Response Quantification

During playback trials, we quantified the behavioral responses of male coal tits (*n* = 20) and green‐baked tits (*n* = 23) to conspecific territorial songs. A response was operationally defined as any flight that brought the male within a 5‐m radius of the nest box or the speaker. Each trial was coded binomially: a positive response (1) was assigned if such an approach occurred, and no response (0) if it did not.

For individuals that responded, we recorded three continuous metrics to assess different dimensions of territorial defense: (1) Time spent (s): total duration spent within the 5‐m zone, indicating sustained engagement with the simulated intruder. (2) Flight frequency: number of distinct flights initiated into the 5‐m zone, reflecting the intensity of the response. (3) Nearest approach distance (m): the minimum distance reached from the speaker, serving as a measure of aggression or approach motivation.

### 2.3 Statistical Analysis

All statistical analyses were performed in R (version 4.4.5; R Core Team 2024). Data manipulation and visualization utilized the *tidyverse* package suite.

### 2.4 Vocal Complexity Analysis

Individual‐level: Differences in syllable repertoire size between species were assessed using a nonparametric Wilcoxon rank‐sum test, following confirmation of non‐normal data distribution via the Shapiro–wilk test.

Population‐level analysis: Syllable usage frequencies were calculated within each species using each syllable type. Acoustic diversity within each species was quantified using Simpson's diversity index (1 − λ) (Jenkins 1978; Podos and Nowicki 2004) computed with the *vegan* package. Pairwise similarities among individuals within each species were assessed using Jaccard similarity coefficients (Real and Vargas 1996; Chao et al. 2005).

### 2.5 Behavioral Response Analysis

We employed Generalized Linear Mixed Models (GLMMs) fitted with the *lme4* package to analyze behavioral responses. For the binary response variable (approach yes/no), a GLMM with a binomial distribution and logit link function was used for both species.

For coal tits, which showed adequate response rates, we additionally fitted separate GLMMs to the three continuous behavioral metrics: time spent (Gaussian distribution), nearest approach distance (Gaussian), and flight frequency (Poisson distribution).

In all GLMMs, stimulus type (Familiar neighbor/Stranger) was included as a fixed effect, and Nest ID was included as a random intercept to control for repeated measures and individual‐specific variation. This modeling approach also accounts for the inherent variability in song‐type sequence and composition present in our natural playback stimuli.

For green‐baked tits, the limited response rate and lack of paired observations prevented the application of robust statistical modeling of the continuous variables. These data are therefore presented descriptively, with distribution visualized using boxplots overlaid with individual data points. The significance threshold was set at α = 0.05 for all inferential tests.

## 3 Results

### 3.1 Vocal Complexity Analysis of Coal Tits and Green‐Backed Tits

#### 3.1.1 Individual Repertoire Size Comparison

**FIGURE 3:**
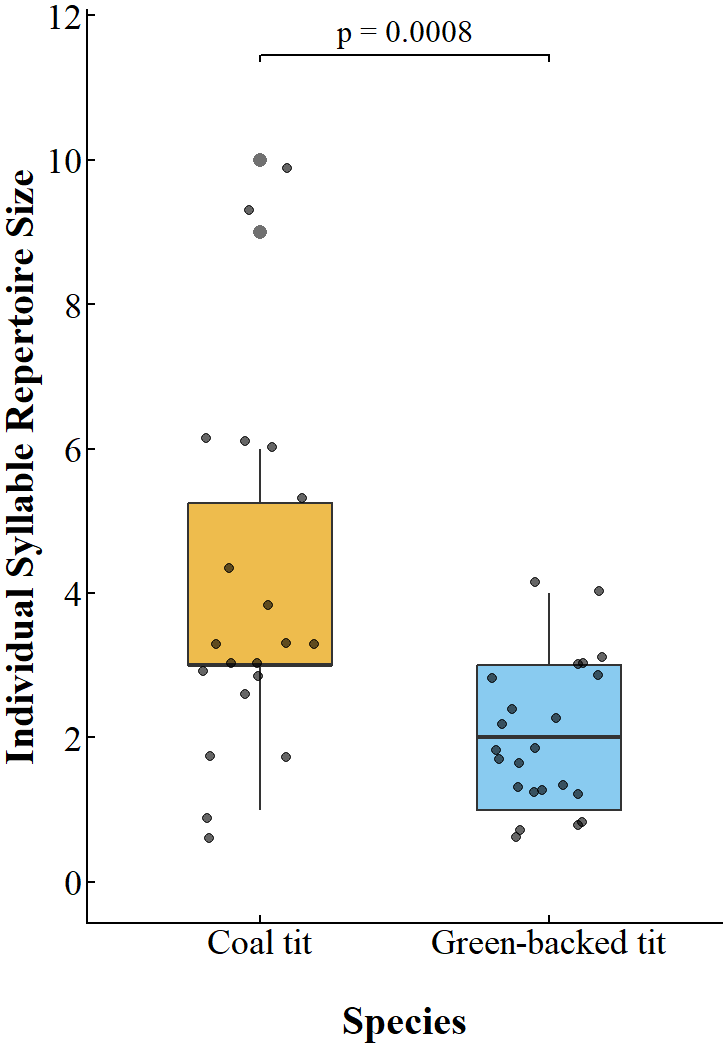
Comparison of individual syllable repertoire size between coal tits and green‐backed tits. Box plots depict the interquartile range (IQR; boxes), median (horizontal line), 1.5 × IQR whiskers, and raw data points (dots). A Wilcoxon rank‐sum test indicated a statistically significant difference between species (*p* = 0.0008).

The individual syllable repertoire size of coal tits (mean ± SD: 4.45 ± 2.50, range 1–10) was significantly larger than that of green‐backed tits (mean ± SD: 1.95 ± 0.90, range 1–4) (Wilcoxon rank‐sum test: W = 364, *p* = 0.0008, Figure 3).

#### 3.1.2 Population‐Level Syllable Usage Patterns

**FIGURE 4:**
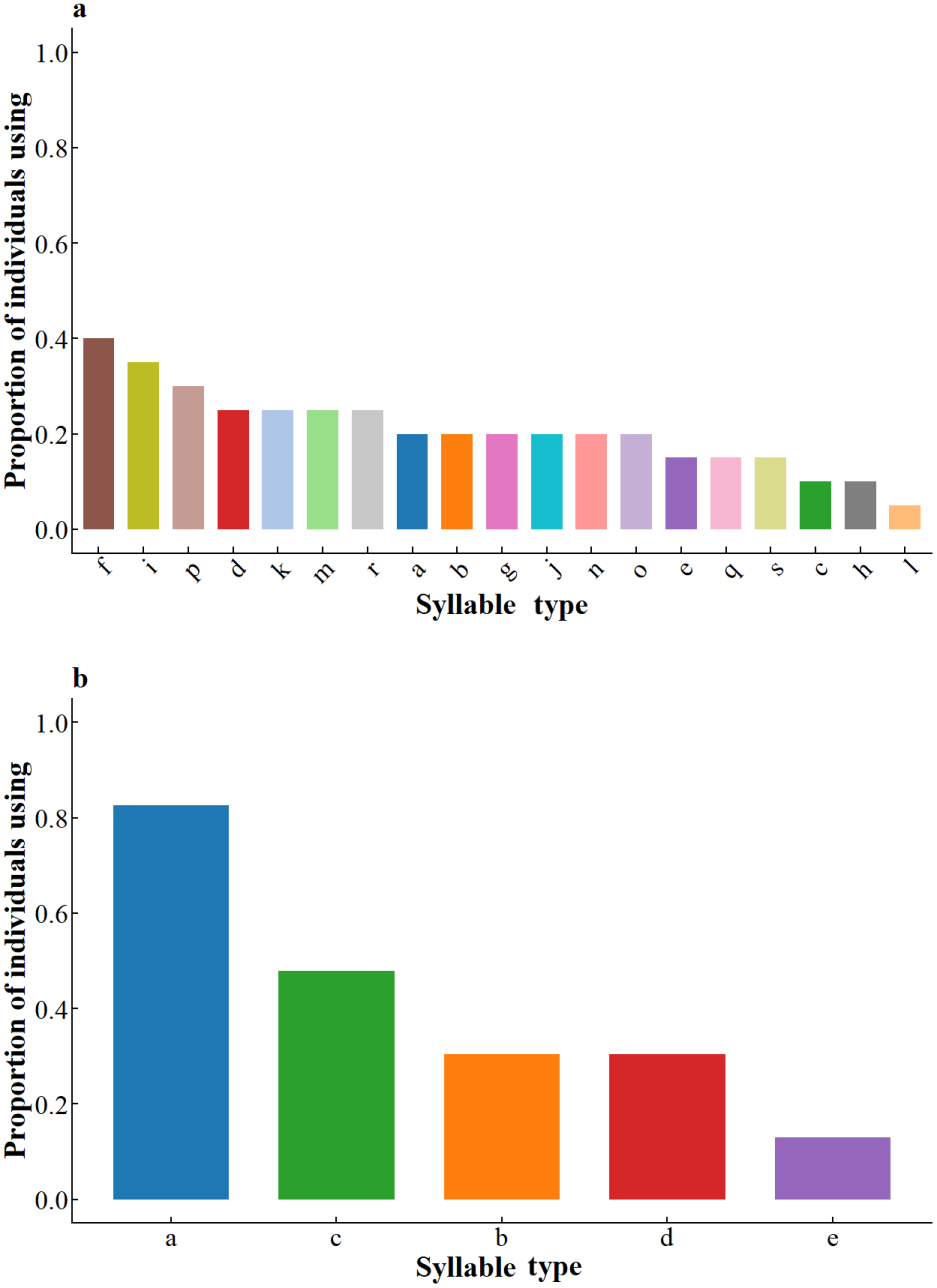
Population‐level syllable usage frequency in coal tits and green‐backed tits. (a) Coal tits employed 19 distinct syllable types (a–s), with types “a,” “f,” and “d” being the most prevalent (each used by 35%–40% of individuals). (b) Green‐backed tits used 5 syllable types (a–e); syllable “a” occurred most frequently (90.9% of individuals), followed by “c” (40.9%) and “d” (27.3%). Bars indicate the proportion of individuals using each syllable type, with distinct colors denoting different syllables.

At population level, coal tit exhibited greater acoustic diversity, utilizing 19 types defined as above (a–s), while green‐backed tits use only 5 types (a–e). Syllable usage frequency distribution revealed that in coal tits, syllables “a,” “f,” and “d” were most prevalent, each used by 35%–40% individuals (Figure 4a). In contrast, green‐backed tit showed high frequency of syllable “a” (used by 90.9% of individuals), followed by “c” (40.9%) and “d” (27.3%) (Figure 4b).

#### 3.1.3 Pairwise Similarity Among Individuals

**FIGURE 5:**
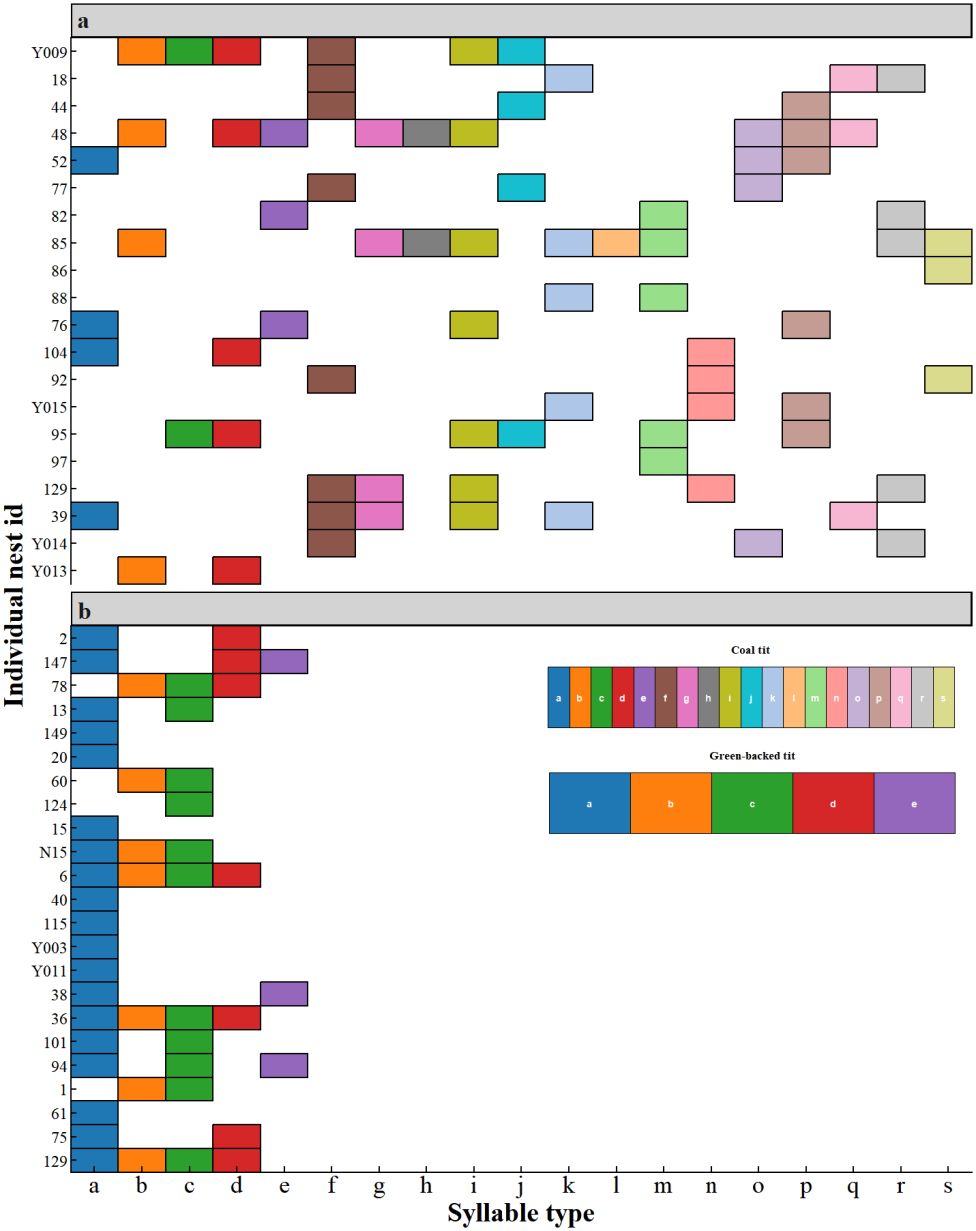
Individual syllable composition and vocal distinctiveness in coal tits and green‐backed tits. (a, b) Binary matrices indicating the presence (colored cells) or absence (white cells) of each syllable type for every individual. Individuals (nest ids) are shown on the y‐axis and syllable types on the x‐axis. Jaccard similarity analysis revealed lower mean similarity among coal tits (0.17 ± 0.14) than among green‐backed tits (0.43 ± 0.19), indicating greater individual distinctiveness in the syllable repertoires of coal tits.

Jaccard similarity analysis revealed lower average similarity among coal tit individuals (mean ± SD: 0.17 ± 0.14) compared to green‐backed tits (mean ± SD: 0.43 ± 0.19), indicating greater individual distinctiveness in the vocal repertoire of coal tits (Figure 5).

#### 3.1.4 Pronounced Interspecific Differences in Response to Conspecific Song Playback

**FIGURE 6:**
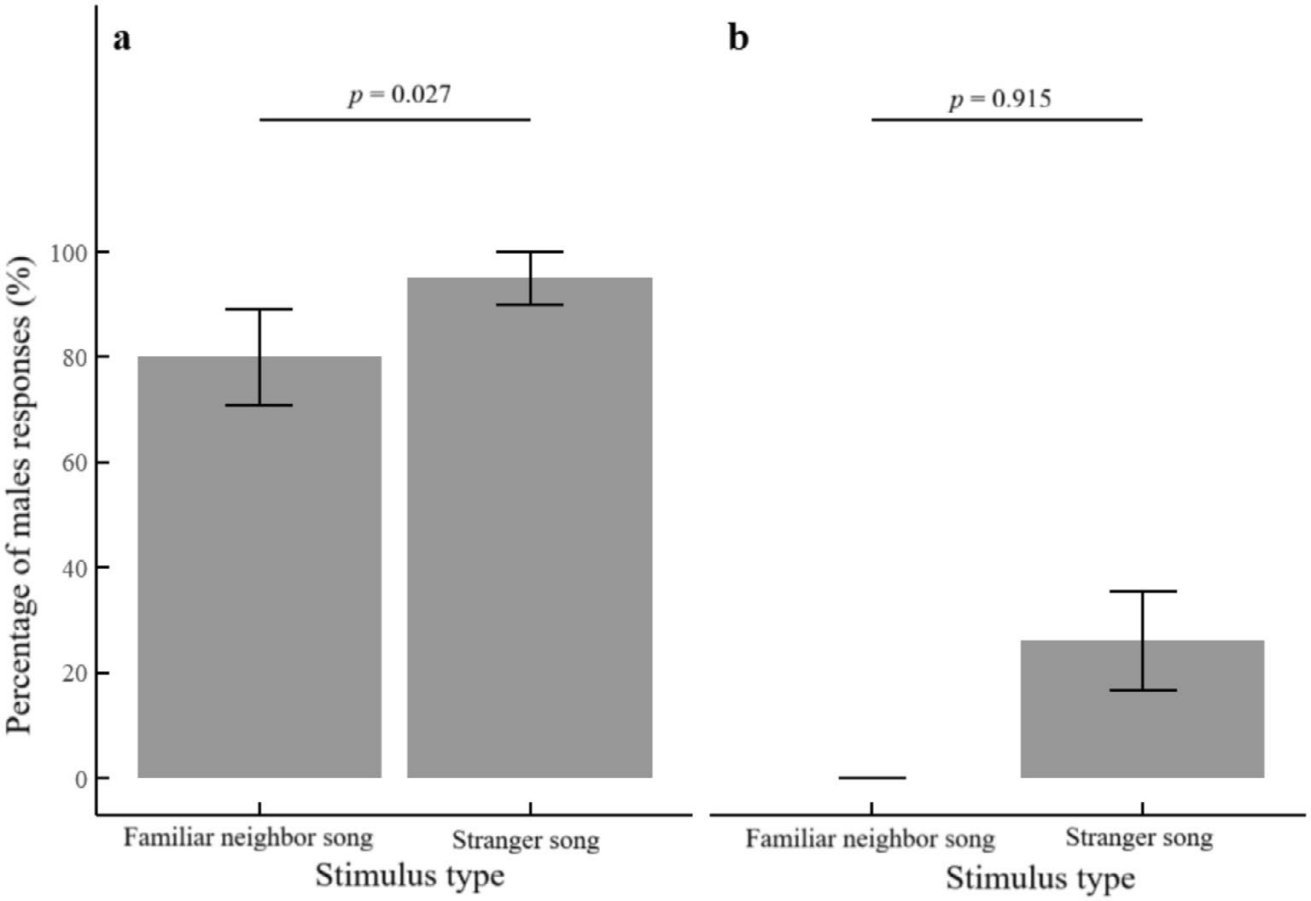
Species‐specific response patterns to conspecific song playbacks. (a) Coal tits significantly discriminated between familiar neighbor and stranger songs (GLMM: *Z* = 2.21, *p* = 0.027), with 95% of males responding to strangers compared to 80% to neighbor songs. (b) Green‐backed tits exhibited low and undifferentiated responses rates (27.3% to strangers, 0% to neighbors; GLMM: *Z* = 0.107, *p* = 0.915). Bars represent mean response percentages of males responding ± SEM The x‐axis indicates stimulus type, and the y‐axis shows the percentage of males that responded.

Playback experiments revealed pronounced interspecific differences in the territorial response strategies of male coal tits and green‐backed tits. Coal tits exhibited high and differential responsiveness: 95% (19/20) of individuals responded to stranger songs and 80% (16/20) to familiar neighbor songs. A binominal GLMM confirmed a significant effect of stimulus type on response probability in this species (*β* = 15.71 ± 7.12 SE, *z* = 2.21, *p* = 0.027, Figure 6a).

In contrast, green‐backed tits showed minimal and undifferentiated responses. Only 26.1% (6/23) respond to stranger songs, and none (0/23) respond to familiar neighbor songs; the GLMM indicated no effect of stimulus type (*β* = 30.78 ± 287.49 SE, *z* = 0.107, *p* = 0.915, Figure 6b).

### 3.2 Coal Tits Display Stronger Territorial Aggression Toward Stranger Songs

**FIGURE 7:**
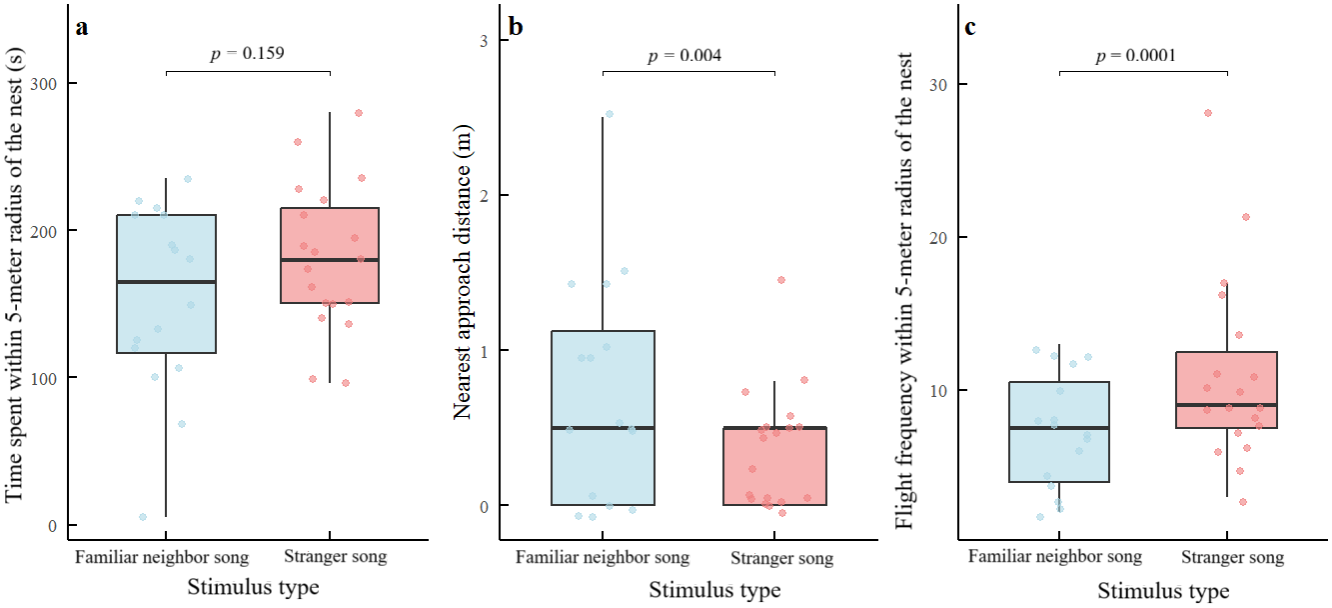
Behavioral responses of coal tits to song playbacks across three metrics of the territorial defense. (a) Time spent within 5‐m radius of the nest box, (b) Nearest approach distance to the speaker, and (c) Flight frequency within 5‐m radius of the nest box. Statistical comparisons (stranger vs. neighbor songs) revealed: Significantly higher flight frequency within 5‐m radius of the nest box (*z* = 3.89, *p* = 0.0001) and shorter nearest approach distance to the speaker (*t* = −3.34, *p* = 0.004), but no significant difference in time spent near the nest (*t* = 1.44, *p* = 0.159). Data are presented as boxplots (medians, interquartile ranges) with overlaid individual data points.

Detailed analysis of three metrics in coal tits (restricted to responding individuals) revealed differentiated aggression. While time spent within 5 m of the nest did not differ between stimuli (*β =* 27.69 ± 19.21 SE, *t* = 1.44, *p* = 0.159, Figure 7a), males approached the speaker more closely during stranger playbacks (*β* = −0.42 ± 0.13 SE, *t* = −3.34, *p* = 0.004, Figure 7b), and performed significantly more flights (*β* = 0.45 ± 0.12 SE, *z* = 3.89, *p* = 0.0001, Figure 7c), indicating heightened vigilance or aggression toward strangers.

### 3.3 Green‐Backed Tits Show Minimal and Undeferential Responsiveness

**FIGURE 8:**
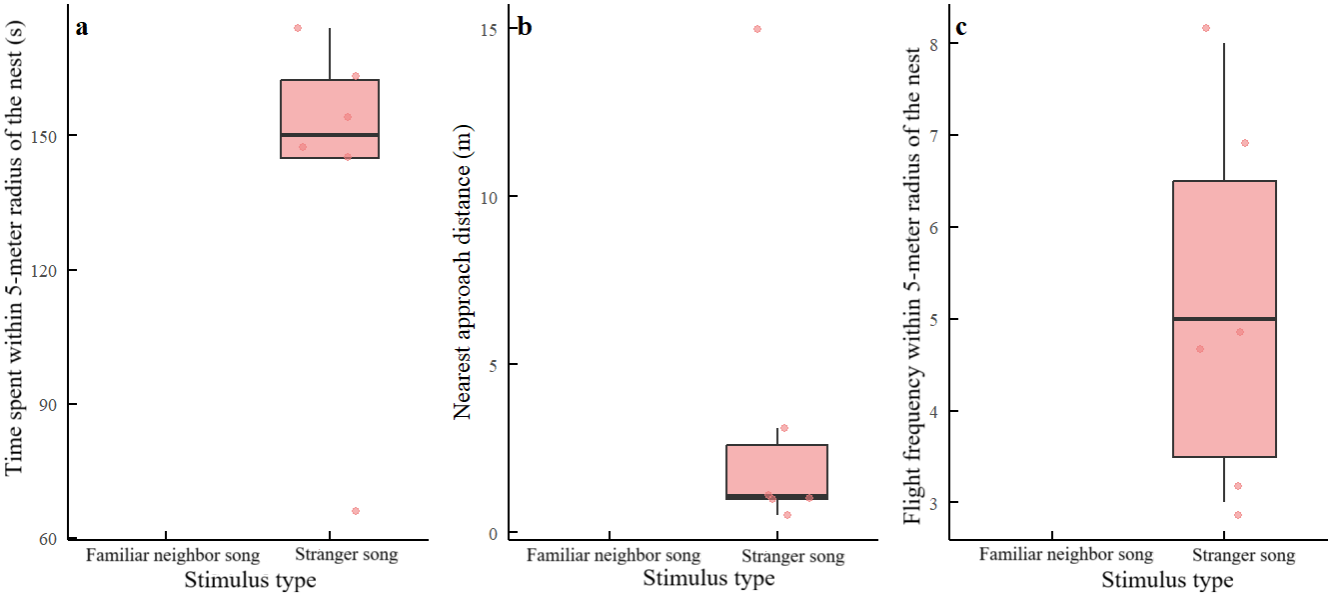
Absence of neighbor–stranger discrimination in green‐backed tits across the three behavioral metrics. (a) Time spent within a 5‐m radius of the nest box, (b) Nearest approach distance to the speaker, or (c) Flight frequency within 5‐m radius of the nest box. No significant differences were detected between familiar neighbor and stranger song playbacks in any of the measured responses, indicating a lack of discrimination. Boxplots show overlapping distributions with considerable within‐group variation. Data are presented descriptively due to sample size constraints precluding formal inferential testing.

Owing to the near‐absence of response to familiar neighbor songs (0/23) and the low response rate to the stranger songs (6/23), no within‐species paired comparison of continuous behavioral metrics was feasible for green‐backed tits. The available data from six individuals that respond to strangers are presented descriptively (Figure 8a–c). The overall pattern of extremely low and undifferentiated response probabilities indicates that: unlike coal tits, male green‐backed tits do not modulate territorial behavior based on vocal familiarity in a manner consistent with the “dear enemy” effect.

## 4 Discussion

Our comparative study reveals a clear interspecific divergence between coal tits and green‐backed tits in both song repertoire architecture and behavioral responses to simulated territorial intruders. Coal tits, possessing large, complex, and individually variable repertoires, exhibited a robust “dear enemy” effect, discriminating strongly between neighbors and strangers. In contrast, green‐backed tits, with their extremely limited and highly stereotyped repertoires, showed uniformly low and undifferentiated aggression. This parallel variation in signal complexity and social discrimination ability between sympatric, ecologically similar congeners provides a compelling model system for investigating how vocal production constraints may shape recognition capabilities (Gallego‐Abenza et al. 2025), and underscores that the presence of NSD cannot be predicted by repertoire size alone (as evidenced in species with simple songs like the blue‐headed wood‐dove *Turtur brehmeri* , Niśkiewicz et al. 2024, and the rufous hornero, Amorim et al. 2023).

### 4.1 Inter‐Specific Divergence in Response Patterns: Implications of Vocal Complexity for Individual Recognition

Our study reveals a profound interspecific divergence in territorial response strategies, which is underpinned by fundamental differences in vocal complexity. Coal tits demonstrated robust discrimination consistent with the dear‐enemy effect, exhibiting significantly heightened aggression (increased flight frequency and closer approach distances) toward stranger songs. This aligns with the strategy of minimizing energetic costs by reducing aggression toward familiar neighbors while focusing on potential threats from strangers (Temeles 1994). In contrast, green‐backed tits showed universally low and undifferentiated responsiveness to both neighbor and stranger playbacks, indicating an absence of both dear‐enemy and nasty‐neighbor effects and suggesting fundamentally different strategies for social threat perception and energy allocation.

The stark contrast in song repertoire complexity provides a mechanistic basis for this behavioral divergence. Coal tits possess a complex and variable repertoire, which likely furnishes a rich “acoustic palette” for encoding unique individual signatures, thereby facilitating neighbor recognition and the threat‐sensitive assessment central to the dear‐enemy effect (Moser‐Purdy and Mennill 2016). Conversely, the minimal, stereotyped repertoire of green‐backed tits, coupled with high acoustic similarity among individuals, may severely constrain the acoustic space for generating readily distinguishable signatures. It is important to note that a small repertoire does not universally preclude neighbor–stranger discrimination, as species like the ortolan bunting ( *Emberiza hortulana* ) can rely on subtle within‐song‐type variation, such as individual differences in the frequency of initial phrases, to achieve recognition (Osiejuk 2014). Therefore, the constraint in green‐backed tits likely arises not merely from repertoire size, but from a potential lack of sufficient and stable individual‐specific acoustic variation (e.g., in spectral or temporal fine structure) within their highly convergent song types, or from a perceptual system not primed to use such cues if they exist.

We emphasize that a small repertoire size does not universally preclude neighbor‐stranger discrimination. Species such as the blue‐headed wood‐dove can achieve fine‐scale recognition using subtle spectrotemporal patterns within simple phrases (Niśkiewicz et al. 2024). Therefore, the constraint in green‐backed tits likely arises from the specific combination of an exceptionally small repertoire and high interindividual vocal convergence. In this context, the inability to reliably distinguish individuals may render a default low‐response strategy an energetically conservative adaptation (Dutour et al. 2024), consistent with a nuanced threat hypothesis where effective assessment is contingent upon prior reliable discrimination (Temeles 1994). Future studies could investigate whether environmental challenges to signal assessment, such as anthropogenic noise, disproportionately impair species like the green‐backed tit that already operate with minimal vocal individuality (Grabarczyk and Gill 2019). In summary, our findings underscore that the capacity for vocal‐mediated individual recognition must be understood within the framework of a species‐specific acoustic system, where the interaction between repertoire simplicity and high vocal convergence can impose a functional constraint on fine‐scale social recognition.

### 4.2 Behavioral Divergence as an Adaptation to Species‐Specific Ecological Constraints

The observed behavioral divergence likely reflects evolved strategies shaped by distinct ecological pressures. For coal tits, a complex vocal repertoire supports efficient territory defense through individual recognition, allowing residents to modulate aggression according to threat level—a strategy that minimizes energetic costs in stable social environments (Briefer, Rybak, and Aubin 2008; Briefer, Aubin, et al. 2008). In such contexts, vocal signaling provides an energetically economical means of maintaining territory boundaries (Jin et al. 2021). Recent work also suggests that phylogenetic relatedness can outweigh acoustic similarity in guiding responses to unfamiliar calls (Sun et al. 2025), a factor that may further contribute to the species' differential response patterns.

In contrast, the minimal song complexity and low responsiveness of green‐backed tits may represent an alternative evolutionary pathway. In ecological settings where territory turnover is high, neighbors are transient, or the benefits of fine‐scale discrimination are low, investing in a complex individual‐recognition system may offer limited returns. Under such conditions, a default low‐response strategy can become adaptive, conserving energy that would otherwise be spent on frequent, undifferentiated territorial challenges (Rossetto and Laiolo 2024). This “minimal response” tactic may reflect a tradeoff wherein the costs of maintaining a sophisticated recognition system outweigh the benefits, favoring instead a strategy of general vigilance or reliance on nonvocal cues.

Together, these patterns highlight how vocal behavior and territorial strategies are tuned to species‐specific ecological realities. Future studies that quantify local population stability, neighbor tenure, and the energetic costs of territorial defense in these tit species could further clarify the ecological drivers underpinning their divergent evolutionary solutions.

### 4.3 Limitations and Future Research Directions

While our comparative approach provides insights into the link between song complexity and social recognition, several limitations should be noted, which also delineate clear paths for future research.

A primary limitation is the absence of a positive control stimulus (e.g., an intense intrusion call) to independently verify the territorial motivation of all subjects during testing. Although the robust, discrimination‐based responses of coal tits confirm the general efficacy of our protocol, we cannot definitively rule out that the uniformly low reactivity of green‐backed tits was influenced by transient motivational states, in addition to potential perceptual constraints. Future playback studies should incorporate positive controls to conclusively disentangle motivational effects from sensory‐cognitive limitations.

The sample size for green‐backed tit songs, while sufficient to establish their small repertoire size, limited more fine‐grained acoustic analyses. A comprehensive quantification of within‐species variability and the precise acoustic features encoding individual identity was thus not feasible.

Although we compared sympatric congeners to control for major ecological differences, unmeasured variables such as fine‐scale territory turnover or historical competition could influence NSD strategies. Furthermore, our two‐species comparison limits broader phylogenetic generalizations about the evolution of recognition systems.

Our experiments were conducted during a stable breeding phase (incubation/early nestling period). The expression of the dear enemy effect can be plastic across seasons; thus, behavioral dynamics during territory establishment or late chick‐rearing stages remain unexplored here.

To address these limitations and advance the field, we propose the following research directions: Future playback studies should incorporate positive controls to conclusively disentangle motivational effects from sensory‐cognitive limitations. High‐resolution acoustic analyses on larger samples are needed to quantitatively assess how individuality is encoded in species with vastly different repertoire architectures, addressing gaps in individual vocal recognition research (Carlson, Healy, and Templeton 2020; Carlson, Kelly, and Couzin 2020). Expanding comparisons across more phylogenetically diverse species pairs will help separate the effects of vocal learning from other evolutionary drivers. The role of phylogenetic relatedness in heterospecific signal perception also warrants further investigation (Sun et al. 2025). Longitudinal playback experiments across different breeding phases will clarify the temporal plasticity of neighbor–stranger discrimination (Briefer, Rybak, and Aubin 2008; Jin et al. 2021). Additionally, investigating the roles of song learning and cultural transmission could provide deeper mechanistic insights into the development of both vocal diversity and perceptual capabilities (Dutour et al. 2024).

## 5 Conclusion

Our study demonstrates the capacity for neighbor‐stranger discrimination is not universal and is closely tied to a species' vocal architecture. We show that coal tits, possessing large and complex song repertoires, exhibit a robust “dear enemy” effect, aligning with models of threat‐sensitive behavioral allocation (Temeles 1994; Salis et al. 2025). In contrast, green‐backed tits, with their limited repertoires, showed undifferentiated, low intensity responses to both neighbors and strangers. This parallel divergence in signal complexity and social discrimination between sympatric congeners suggests that severe constraints on vocal production can limit the acoustic potential for individual recognition, thereby shaping alternative territorial strategies. This study highlights bioacoustics constraints in shaping social behavior and underscores the importance of integrating vocal production mechanisms into broader behavioral ecological models of communication and conflict resolution (Dutour et al. 2024). Furthermore, our findings contribute to a growing framework that considers potential phylogenetic influences on signal perception (Sun et al. 2025) and reliable information sharing importance in mixed‐species systems (Carlson, Healy, and Templeton 2020; Carlson, Kelly, and Couzin 2020) as key drivers in the evolution of complex signaling systems.

## Author Contributions

**Lin Zhao:** data curation (lead), formal analysis (lead), investigation (equal), methodology (equal), visualization (equal), writing – original draft (equal). **Fangfang Zhang:** investigation (equal), methodology (equal). **Jianping Liu:** resources (equal), supervision (equal), writing – review and editing (equal). **Wei Liang:** conceptualization (lead), funding acquisition (equal), supervision (equal), validation (equal), writing – review and editing (equal).

## Funding

This work was supported by the National Natural Science Foundation of China (no. 32470513 to WL) and 2023 Ningxia Hui Autonomous Region Youth Science and Technology Support Talent Training Project.

## Ethics Statement

The experiments comply with the current laws of China, where they were performed. Experimental procedures were in agreement with the Animal Research Ethics Committee of Hainan Provincial Education Centre for Ecology and Environment, Hainan Normal University (nos. HNECEE‐2014‐005 and HNECEE‐2024‐003).

## Conflicts of Interest

The authors declare no conflicts of interest.

## Supporting information


**Table S1:** Data for behavioral response of coal and green‐backed tits to playback experiments.

**Table S2:** Data for song type coded of two coders in coal and green‐backed tits.

**Table S3:** Data for organized playback song in coal and green‐backed tits.

## Data Availability

Data used for this study are provided as Supporting Information (Tables [Link], [Link]) and can be found at https://figshare.com/s/3502581cddf7d0f9f79a (doi: 10.6084/m9.figshare.30254089).
